# The ASAMET trial: a randomized, phase II, double-blind, placebo-controlled, multicenter, 2 × 2 factorial biomarker study of tertiary prevention with low-dose aspirin and metformin in stage I-III colorectal cancer patients

**DOI:** 10.1186/s12885-018-5126-7

**Published:** 2018-12-04

**Authors:** Marilena Petrera, Laura Paleari, Matteo Clavarezza, Matteo Puntoni, Silvia Caviglia, Irene Maria Briata, Massimo Oppezzi, Eva Mihajlovic Mislej, Borut Stabuc, Michael Gnant, Thomas Bachleitner-Hofmann, Wilfried Roth, Dominique Scherer, Walter-E. Haefeli, Cornelia M. Ulrich, Andrea DeCensi

**Affiliations:** 1 Division of Medical Oncology, E.O. Ospedali Galliera, Mura delle Cappuccine 14, 16128 Genoa, Italy; 2 A.Li.Sa., Public Health Agency, Liguria Region, Italy; 3 Clinical trial office, Scientific directorate, E.O. Ospedali Galliera, Genoa, Italy; 4 Department of Gastroenterology and Digestive Endoscopy, E.O. Ospedali Galliera, Genoa, Italy; 5 Clinical Department of Gastroenterology, University Medical Center Ljubljana, Ljubljana, Slovenia; 6 Department of Surgery and Comprehensive Cancer Center and Austrian Breast and Colorectal Cancer Study Group (ABCSG), Vienna, Austria; 7 Institute of Pathology University Medical Center Mainz, Mainz, Germany; 8 Institute of Medical Biometry and Informatics, University of Heidelberg, Heidelberg, Germany; 9 Department of Clinical Pharmacology and Pharmacoepidemiology, Heidelberg University Hospital, Heidelberg, Germany; 10 Huntsman Cancer Institute, Salt Lake City, UT USA; 11 Wolfson Institute of Preventive Medicine, Queen Mary University of London, London, UK

**Keywords:** Colorectal cancer, Aspirin, Metformin, Biomarkers, Chemoprevention, Tertiary prevention

## Abstract

**Background:**

Epidemiological studies and cardiovascular prevention trials have shown that low-dose aspirin can reduce colorectal cancer (CRC) incidence and mortality, including inhibition of distant metastases. Metformin has also been associated with decreased colon adenoma recurrence in clinical trials and lower CRC incidence and mortality in epidemiological studies in diabetics. While both drugs have been tested as single agents, their combination has not been tested in cancer prevention trials.

**Methods/design:**

This is a randomized, placebo-controlled, double-blind, 2 × 2 biomarker trial of aspirin and metformin to test the activity of either agent alone and the potential synergism of their combination on a set of surrogate biomarkers of colorectal carcinogenesis. After surgery, 160 patients with stage I-III CRC are randomly assigned in a four-arm trial to either aspirin (100 mg day), metformin (850 mg *bis in die)*, their combination, or placebo for one year. The primary endpoint biomarker is the change of IHC expression of nuclear factor kappa-B (NFκB) in the unaffected mucosa of proximal and distal colon obtained by multiple biopsies in two paired colonoscopies one year apart. Additional biomarkers will include: 1) the measurement of circulating IL-6, CRP and VEGF; 2) the IHC expression of tissue pS6K, p53, beta-catenin, PI3K; 3) the associations of genetic markers with treatment response as assessed by next generation sequencing of primary tumors; 4) the genomic profile of candidate genes, pathways, and overall genomic patterns in tissue biopsies by genome wide gene expression arrays; and 5) the evaluation of adenoma occurrence at 1 year.

**Discussion:**

A favorable biomarker modulation by aspirin and metformin may provide important clues for a subsequent phase III adjuvant trial aimed at preventing second primary cancer, delaying recurrence and improving prognosis in patients with CRC.

**Trial registration:**

EudraCT Number: 2015–004824-77; ClinicalTrial.gov Identifier: NCT03047837. Registered on February 1, 2017.

## Background

Colorectal cancer is the second most frequent cause of cancer-related death in Western Europe. Despite advances in surgical and adjuvant treatment over the past two decades, survival remains poor, with a 5-year survival well below 60% in patients undergoing resection with curative intent [1, 2]. Recent results of EUROCARE-5, a retrospective observational study based on data from 107 cancer registries for more than 10 million patients with cancer diagnosed up to 2007 and followed up to 2008, provide clear evidence that effective treatment of colorectal cancer (CRC) is currently an unmet medical need in Europe [3]. For CRC, the European mean age-standardized 5-year survival was 57.0% (95%CI 56.8–57.3), with negligible differences between the sexes. Given that the current standard adjuvant therapy of CRC has not improved for the last 10 years, there is a clear need for a preventive intervention aimed at assessing the effectiveness of innovative and low toxicity interventions designed to prevent tumor recurrence and/or second cancer. Aspirin and metformin, two common, safe and inexpensive drugs with a broad spectrum of preventive effects on several target systems, have recently shown a preventive potential (both in primary and tertiary prevention) against several cancers, including CRC.

Meta-analyses of randomized clinical trials for the prevention of vascular disease indicate that daily aspirin (75 mg upwards) reduces cancer incidence and mortality. These effects are particularly evident for CRC where a 30–40% reduction in incidence and mortality have been observed [4, 5]. The risk of developing distant metastasis was also reduced in aspirin users (HR 0.45; 95% CI 0.28–0.72), with a further lowering risk for CRC patients (HR 0.26, 95 %CI, 0.11–0.57) [6]. Benefits were already evident as early as after 2 years from treatment initiation. These shorter-term effects of aspirin on distant metastasis are consistent with experimental evidence in animals due to platelets involvement in blood-borne metastases [7]. Data obtained from the Nurses’ Health Study and the Health Professionals Follow-up Study also showed that regular use of aspirin after diagnosis was associated with longer survival among patients with mutated-PIK3CA CRC [8], and our recent meta-analysis suggests that the benefit of post-diagnosis aspirin treatment on overall mortality in CRC may be more marked in PIK3CA mutated tumours [9].

The recognition that the hyper-insulinemic state associated with metabolic syndrome or type II diabetes mellitus is linked with increased cancer risk has led to intensified interest in the potential of various anti-diabetic drugs to prevent cancer [10]. Higher body mass index (BMI) is strongly associated with CRC risk and with high levels of insulin and insulin growth factors (IGFs) [11].

As a first-line diabetic agent, metformin has been associated with reduced cancer incidence and mortality in diabetic patients[12] and CRC patients specifically [13]. In a clinical trial of individuals not taking non-steroidal anti-inflammatory drugs (NSAIDs), a 250 mg/day dose of metformin was sufficient to decrease aberrant crypt foci, suggesting that a lower dose of metformin could play a role in colon cancer prevention in this population [14]. In a recent randomized [15], placebo-controlled, multicenter trial, in patients who underwent resection of single or multiple colorectal adenoma polyps, a low-dose of metformin (250 mg/day) administered for 1 year significantly reduced the risk of total polyps (RR = 0.67; 95% CI: 0.47–0.97) and adenomas (RR = 0.60, 0.39–0.92) compared with placebo.

Several preclinical studies have reported that aspirin and metformin reduced cell proliferation, induced apoptosis, and caused cell cycle arrest targeting common signaling pathways. Both drugs are activators of AMP kinase (AMPK), leading to down-regulation of the mTOR pathway that is crucial to tumor cell metabolism [16, 17]. Moreover, their combination has a striking additive effect on AMPK activation and mTOR inhibition, with increased autophagy in CRC cell lines [18, 19]. The two agents have also been shown to act synergistically at low concentrations (1–5 mmol/L) to inhibit pancreatic cancer cell line growth and increase cell death by inhibiting the phosphorylation of mTOR, S6K, JAK2, and STAT3 [20]. It has been reported that metformin activated AMPK and inhibited mTOR by suppressing NF-κB and CREB [21]. Besides, aspirin has shown a pro-apoptotic activity through modulation of the NFκB pathway, which was particularly evident in CRC cells [22]. NF-kB is not a validated clinical endpoint for CRC but plays a pivotal role in tumor initiation and progression and is known to be constitutively activated in CRC [23].

To date, no clinical trial has ever tested the combination of aspirin and metformin. Our clinical trial aims to assess a potential synergistic interaction on a set of biomarkers associated with colon carcinogenesis, some of which may serve as predictive biomarkers of drug effects. This will be tested on biomarkers expressed in the unaffected colonic mucosa, which are associated with the adenoma carcinoma sequence. The cancerization field effect represents a pre-malignant stage in progression to many cancers, so the use of the patients’ unaffected mucosa for the evaluation of biomarkers in patients with prior CRC may provide an indicator of preventive activity/efficacy [24, 25].

## Methods/design

### Objectives

#### Primary objective

The primary objective is to test the synergistic effect of the combined treatment with low dose aspirin plus metformin given for one year to reduce the ICH expression of NFκB in unaffected colonic tissue in patients with removed CRC. The primary endpoint is the change, defined as the difference between post- and pre-treatment levels, in NFκB expression in the unaffected mucosa of proximal and distal colon obtained by multiple biopsies in two paired colonoscopies one year apart.

#### Secondary objectives

Secondary objectives are:

1. To test the effect of treatment with aspirin and metformin, in combination and independently, on the following secondary endpoints:

- The change in immunohistochemistry (IHC) expression levels of pS6K, p53, β-catenin, PI3K (from unaffected colon biopsy specimens);
- The change in the circulating biomarkers IL-6, CRP, VEGF, and HOMA index [homeostasis model assessment [fasting blood glucose (mmol/L)*insulin (mU/L) / 22.5];
- The gene expression levels of candidate genes (PTGS1-COX1, PTGS2-COX2, VEGF, TNFα, EGFR, NFκB), pathways (mTOR signaling - KEGG04150; NFκB signaling - KEGG04064; VEGF signaling – 04370; FoxO signaling KEGG04068; Regulation of autophagy - KEGG04140), and genome-wide expression profile in unaffected colon biopsy tissue.

2. To define the blood and tissue drug levels of metformin, considering the genetic variability of specific membrane-bound drug transporters which may affect metformin pharmacokinetics and tissue distribution.

3. To genetically characterize the primary CRC using next generation sequencing (NGS) on a panel comprising 180 amplicons of 30 genes (including KRAS, BRAF, NRAS, APC, PIK3CA, TP53, CTNNB1 and EGFR) and determine their association with treatment response.

4. To study the treatment tolerability comparing incidence and grade of toxicities (safety endpoint) among arms (categorized using the Common Terminology Criteria for Adverse Events, CTC-AE version 4.03).

5. To study the serum concentrations of thromboxane B2 (TxB2) as a biomarker of treatment adherence to be correlated with biomarker modulation and toxicity.

6. To evaluate the effect of treatment on psychological variables (distress, anxiety, and depression) and cancer-related fatigue and to explore their interactions with treatment on the endpoint biomarkers.

7. To evaluate the occurrence of adenoma (low, intermediate and/or high grade intraepithelial neoplasia) at baseline and 12 months after randomization according to the Vienna classification of gastrointestinal epithelial neoplasia.

In a patient subset additional analyses are planned with the following secondary objectives:

- Metabolomic analysis: the change of low molecular weight compounds in serum samples (up to 53 analytes) to obtain a signature of response to therapy.
- The 12 month change of microbial composition by sequencing of the 16S rRNA gene of the colorectal microbiota by NGS analysis in colonic tissue and feces.
- The 12 months change in auto-antibody compositions in serum samples (ASMA, AMA, APCA, ANA, ANCA, ASCA, ENA/dsDNA, and TPO) by ELISA and immunoblotting assays.
- To evaluate the interactions between treatment and physical activity, life style and food habits on the endpoint biomarkers.

Moreover, an exploratory objective of the study is to discover analytically (using STEPP statistical analysis, see statistical methods) putative metabolic/molecular and psychological patterns able to define subgroups of patients in which treatment could be more (or less) active (personalized treatment).

### Study design

The study is a randomized phase II, double-blind, double-dummy, placebo-controlled, multicenter, 2 × 2 factorial design clinical trial in cancer patients, aged ≥ 18 ≤ 80 years, with completely resected stage I-III primary CRC within 24 months prior to randomization, regardless of (neo-)adjuvant chemotherapy. A total of 160 patients will be accrued, during an 18-month enrollment period. All patients will participate in up to 28 days screening, during which they will be assessed for eligibility. After screening and verification of eligibility criteria, eligible patients who provide informed consent will be randomly allocated with a ratio of 1:1:1:1 to one of the following 4 arms (40 patients per arm) and treated for one year:

Arm A: placebo aspirin (1 tablet daily, *quaque die*, qd) + placebo metformin (1 tablet *bis in die,* bid).

Arm B: placebo aspirin (1 tablet qd) + active metformin (850 mg metformin, 1 tablet bid).

Arm C: active aspirin (100 mg acetylsalicylic acid, 1 tablet qd) + placebo metformin (1 tablet bid).

Arm D: active aspirin (100 mg, 1 tablet qd) + active metformin (850 mg, 1 tablet bid).

**Fig. 1:**
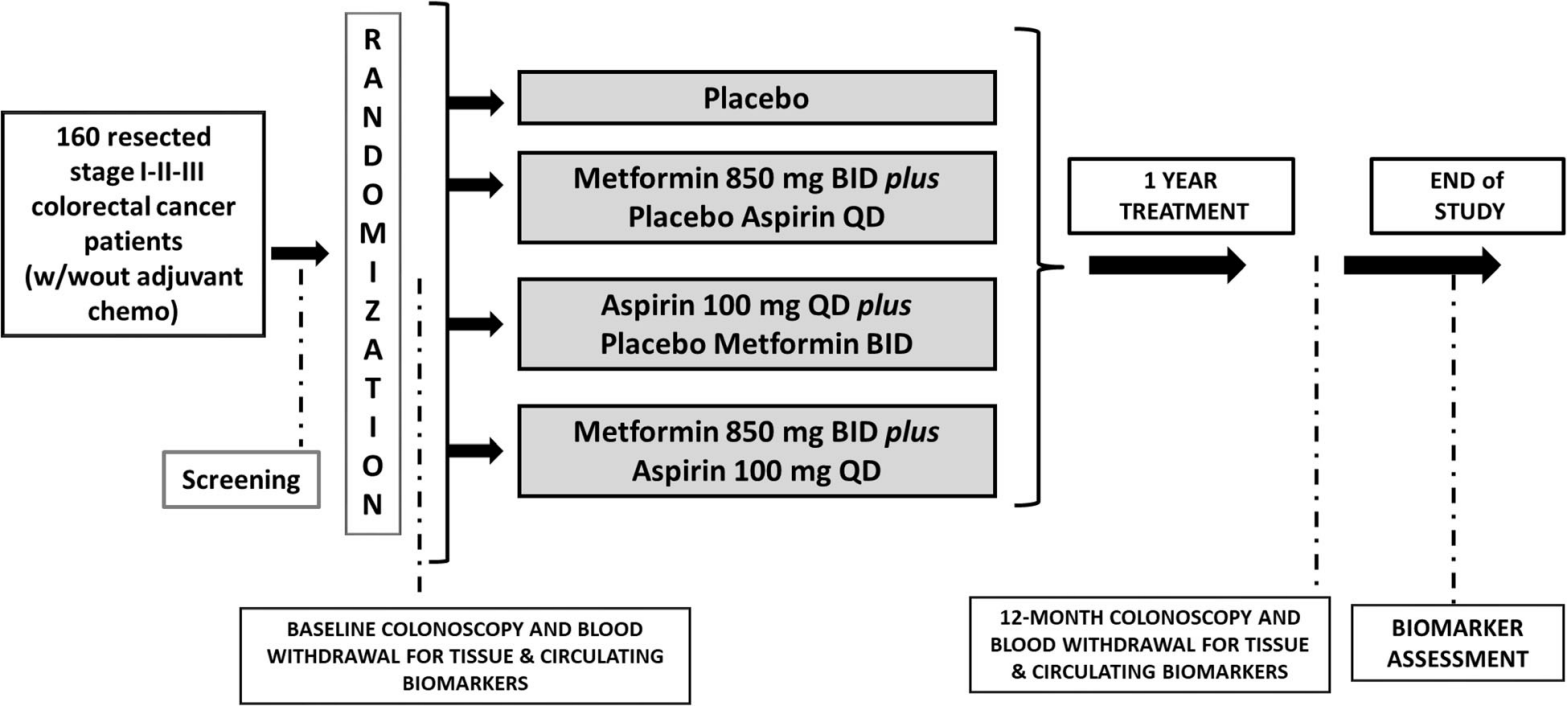
Overall Study Design

Figure 1 illustrates the overall study design. Table 1 presents the inclusion/exclusion criteria.

**Table 1:** Inclusion and exclusion criteria

Inclusion criteria	Exclusion criteria
•Patients aged > 18 and ≤ 80 years. •Patients with completely resected stage I, II, or III primary CRC within 24 months prior to randomization, regardless of (neo-)adjuvant chemotherapy. Patients with pT1 CRC treated with endoscopic polypectomy. •Adjuvant chemotherapy and (neo-)adjuvant radiotherapy terminated at least 3 months before randomization. •ECOG performance status ≤1. •Platelets ≥100 × 10^9/L; •Creatinine clearance estimated with the Cockcroft - Gault formula ≥60 mL/min. Patients with Gault formula ≥30–59 ≤ ml/min are eligible but they will receive a single (evening) tablet of metformin, 850 mg. •AST and ALT ≤2.5 times upper limit of normal (ULN). •Females of childbearing potential/males with partners of childbearing potential participating in the study are to use effective methods of birth control during study participation. Female participants must provide a pregnancy test, according to local/national guidelines. •Able to understand and sign an informed consent (or have a legal representative who is able and willing to do so).	•Patients who are not able to undergo colonoscopy. •Patients who are allergic or intolerant to ibuprofen or naproxen, or who have metformin, or aspirin, or salicylate intolerance or more generalized drug intolerance to NSAIDs. •Any serious medical condition, laboratory abnormality, or psychiatric illness that would prevent the patient from signing or participating in the study and/or comply with study procedures. •Chronic treatment with aspirin or other NSAIDs or metformin or patients who are on current long-term treatment (≥ 4 consecutive weeks) with aspirin, NSAID, COX − 2 inhibitors, or metformin. •Diabetic patients on drug treatment. •Anticoagulant therapy (e.g. dicumarol, heparin, fondaparinux, apixaban, dabigatran etexilate, rivaroxaban) or active current treatment with antiplatelet agents (e.g. off-study aspirin, clopidogrel, prasugrel, ticagrelor, or ticlopidine). •Any other invasive malignancies (with the exclusion of basal cell carcinoma or cutaneous squamous cell carcinoma) diagnosed during the last 5 years before randomization. Past history of any other invasive CRC than the one the patient is currently being treated for. •Alcohol or drug abuse. •Prior history of gastro-intestinal bleeding or hemorrhagic diathesis (e.g. hemophilia). •Erosive-ulcerative lesions in the gastrointestinal tract. •History of erosive gastro-esophageal reflux disease (GERD) or active erosive GERD on gastroscopy. •Concomitant corticosteroid treatment. •Known deficiency of glucose-6-phosphate dehydrogenase (G6PD). •Treatment with another investigational drug < 28 days prior to study entry. •Concurrent participation in a clinical trial with the same endpoints. •History of hemorrhagic stroke. •Lynch Syndrome. •Crohn’s disease or ulcerative colitis. •Pregnant or lactating females. •History of lactic acidosis. •Liver dysfunction including chronic active hepatitis and cirrhosis not compensated. •History of vitamin B12 deficiency or megaloblastic anemia. •Uncontrolled coronary syndrome or symptomatic congestive heart failure (e.g. Class III or IV New York Heart Association’s Functional Classification). •Inability or unwillingness to swallow tablets.

### Study procedures

An overview of “Visit schedule and assessments” is provided in Table 2.

**Table 2:** Visit schedule and assessments

Assessments			Treatment Period				
	Screening^1^	Randomization	Baseline	4 Months	8 Months	12 Months	End of study visit
Informed consent	X						
Inclusion/exclusion criteria	X						
Relevant medical history	X						
Prior medication/therapy of current CRC	X						
Physical exam, vital signs and ECOG performance status	X		X	X	X	X	X
Hematology, biochemistry, and coagulation profile	X^2^		X^2^	X^2^	X^2^	X^2^	
Randomization		X					
Blood collection for biological assessment			X			X	
Blood collection for TxB2/metformin quantification^3^			X	X	X	X	
Feces collection^4^			X			X	
Questionnaires (FACIT, HADS, Distress, IPAQ, and EPIC [4])			X			X	
Colonoscopy and biopsies			X^5^			X^5^	
Adverse events	X^6^		X	X	X	X	X
Concomitant medication			X	X	X	X	
Study treatment supply			X	X	X		
Compliance (via pill count)				X	X	X	

1. Within 28 days after day 1

2. Complete blood count (WBC, white blood cell differential count, RBC, Hb, Ht, MCV, MCH, MCHC, RDW, PLT, and MPV), alanine aminotransferase (ALT), aspartate aminotransferase (AST), creatinine, fasting glucose, triglycerides, and cholesterol. Coagulation profile (coag) includes: (International Normalized Ratio) INR, Activated Partial Thromboplastin Time (APTT), prothrombin time (PT), and fibrinogen. At baseline, hematology, biochemistry, and coagulation profile are not needed if the screening visit is performed within 7 days prior to the baseline visit.

3. TxB2/metformin quantification will be performed at the end of the study

4. Only for Italian patients

5. During the colonoscopies at baseline and at 12 months, the occurrence of adenoma (low, intermediate, and/or high grade intraepithelial neoplasia) as well as the histopathological subtype (e.g. tubular, villous, tubulovillous, sessile serrated, traditional serrated, or hyperplastic) will be determined upon routine histopathological analysis according to local practice (i.e. according to the Vienna classification of gastrointestinal epithelial neoplasia). If at least two adenomas with different grading exist, the highest grade will be considered, e.g. occurrence of two intermediate-grade and one high-grade adenoma would result in a categorization as high-grade

6. Adverse event (trAE) reporting will begin on the date the patient provides informed consent to participate in the study (documentation in the eCRF will only be done for randomized patients) and end 30 days after last study drug dose or at the end-of-study visit, which ever occurs first. During the screening phase, only AEs deemed to be serious (SAEs) and related to protocol and not routinely performed procedures have to be reported

#### Screening visit/ registration

Potential patients will be invited for a detailed discussion of the protocol with the Principal Investigator or a delegate. After obtaining written informed consent, patients will undergo within 28 days prior to first study drug administration (day − 28 to day 1) the following screening procedures: inclusion and exclusion criteria check (Table 1); review of medication history and therapy of current CRC; physical examination: height, weight, measurement of vital signs (including blood pressure, heart rate, and body temperature), and registration of ECOG Performance Status; haematology, biochemistry, and coagulation profile, and a pregnancy test for women with childbearing potential according to local/national guidelines.

#### Baseline

At baseline, all enrolled patients will undergo colonoscopy with biopsies and blood samples for biomarker assessment. At baseline and after 12 months, only the Italian patients will collect feces before colon cleansing in preparation for colonoscopy. Before colonoscopy, patients will complete validated questionnaires to evaluate cancer related fatigue (FACIT-F), anxiety and depression (HADS), cancer-related distress (Distress), physical activity (IPAQ), and eating habits (EPIC- FFQ short version for the Italian patients only). The baseline colonoscopy will be performed between 3 and 24 months after surgery as per standard clinical practice. The occurrence of adenoma (low, intermediate, and/or high grade intraepithelial neoplasia) will be determined using standard clinical practice. In patients treated with adjuvant chemotherapy, baseline colonoscopy will be performed at least 3 months after chemotherapy completion. Pinch biopsies are not standard of care in unremarkable areas of the colonic tissue but have not been associated with increased risk of bleeding or perforation [26, 27]. At the baseline clinic visit, patients will be instructed on the importance of drug compliance and will be provided with an investigational drug kit and a pill diary to be completed daily, signed, and dated. The first drug administration should occur the day after colonoscopy/baseline visit. Concomitant medication and any adverse event (AE) occurring from the screening period will be registered.

#### 4 and 8 month visits

A clinical visit will be performed every 4 months (± 2 weeks) to register vital signs, umbilical waist circumference (UWC), ECOG performance status, adverse events, drug compliance, concomitant medication and smoking habits. Blood samples will be collected for safety tests, metformin and TXB2 quantification in plasma and serum. At each 4 months visit patients will be provided with a new investigational drug kit and compliance evaluation via pill count will be done.

#### 12-month visit

One year (± 2 weeks) after day 1, patients will return to the clinic to repeat colonoscopy and biopsies as well as blood sample collection for safety tests and biomarker assessments. Before colonoscopy, the following procedures will be done: measurement of vital signs; registration of ECOG performance status; update of concomitant medications and procedures; collection of questionnaires; side effect and compliance evaluation via pill count; and for the Italian subset of patient’s feces collection.

#### End of study visit

The end of study visit will be performed 30 days after last study agent dose per protocol (Visit 12 months + 30 days). For patients who will end treatment early, a visit will be performed 30 days after the last intake of study agent. All patients will undergo a physical examination (weight, UWC, and measurement of vital signs), registration of ECOG performance status and side effect evaluation.

### Colonoscopy and pinch biopsies clinical procedures

All enrolled patients will undergo colonoscopy with biopsies at baseline and after 12 months of treatment. Colonic biopsies will be collected both in the right and the left colon (6 specimens per site) in order to study the pharmacokinetic and pharmacodynamic properties of the drugs and to verify if there is a different preventive effect in the right versus left colon according to a different anatomic gradient of mutational status [28]. In patients who received right colectomy, 6 biopsies will be taken at the level of the *cul de sac* and 6 biopsies 2 cm above the third Houston valve. In patients operated for a left-side CRC, 6 biopsies will be collected near the ileo-cecal valve and 6 biopsies 2 cm above the third Houston valve or 2 cm above the anastomosis in patients operated for rectal cancer.

### Trial organization

The ASAMET trial is designed and coordinated by the Medical Oncology Unit at E.O. Ospedali Galliera, Genoa, Italy. Statistical analysis of the main clinical trial will be performed at the Clinical trial office of the coordinating center (Galliera). The Austrian Breast and Colorectal Cancer Study Group is responsible for the Clinical Data Management System of the entire trial (including programming of the clinical database, i.e., electronic Case Report Forms for web-based data entry) and for the randomization system. The enrollment of this multicenter project will be conducted at the following sites: Galliera Hospital, Italy; University Medical Centre Ljubljana, Slovenia; Medical University of Vienna, Medical University Innsbruck and Hospital Wels-Grieskirchen (Klinikum Wels-Grieskirchen GmbH), Austria. The immunohistochemical assessments will be performed by the Institute of Pathology, University Medical Center Mainz. The pharmacokinetic assays and the circulating and genomic biomarkers assessments will be performed at the Department of Clinical Pharmacology and Pharmacoepidemiology and the Statistical Genetics Group Institute of Medical Biometry and Informatics, University Hospital Heidelberg, Germany, respectively.

### Randomization and blinding

The assignment of patients to treatment arms is performed using the centralized randomization system ‘Randomizer®’, which is maintained by the Institute of Medical Statistics and Informatics at the Medical University of Vienna. The Randomizer provides a self-serve, easy-to-use, secure, and 24 h-a-day randomization service for multi-center clinical trials that runs exclusively on the Internet. Randomization is performed using a permuted randomized blocks design. Stratification factors are the center of enrollment and (neo-)adjuvant chemotherapy (no versus yes). The confirmation of randomization is automatically generated by the system and sent back to the specified recipient via e-mail. Details for the randomization procedure are provided in a manual stored in the Trial Master File/Site Study File of each enrolling center. The study staff will dispense the treatment to the patients, during the baseline visit, based on the assigned randomization number/kit code. None of the staff interacting with patients will know the link between kit code and actual treatment. The code that identifies the treatment will be kept by the statistician at the central data management site.

Unblinding is not expected to occur until all patients complete their intervention and data entry is complete. If deemed medically necessary, study treatments may be unblinded by the Principal Investigator in consultation with the Project Coordinator in the event of a serious adverse event.

### Safety profile and monitoring

In this study, all AE encountered during the clinical study will be reported on case report forms. In case of a serious adverse event (SAE), the investigator has a legal requirement to inform the coordinating center office within 24 h via the SAE report form. The coordinating center is responsible for the management of the safety reporting according to European regulation and guidelines. All observed AEs will be evaluated and graded according to the CTCAE, version 4.03. All observed SAEs will be discussed by an independent data safety monitoring board.

Patients will be asked to maintain the full dose throughout the treatment period. However, in case of grade 2 or higher toxicity, dose modification as per protocol will be applied and recorded. The dose will be changed according to the potential relationship to study drugs.

The incidence of metformin-related diarrhea in patients with CRC after surgery is unknown and will therefore be closely monitored. Possible dose modifications in the initial weeks of treatment may be envisioned if the incidence of this AE turns out to be high.

A Data and Safety Monitoring Board (DSMB) will review the progress of the clinical trial and monitor participant safety. During their scheduled meetings, the DSMB will review clinical data, and meeting reports or recommendations will be forwarded to the PIs.

### Sample size and power consideration

The sample size is 40 patients per arm, resulting in a total of 160 patients to be enrolled. Results from our previous clinical trial [29] showed a mean change (standard deviation, SD) of the difference between post- and pre- treatment levels in NFκB equal to + 5% (25%) in the placebo arm versus − 13.5% (25%) in the active (allopurinol) arm. This is equivalent to a − 18.5% absolute difference between treatment arms. Table 3 illustrates the hypothesized treatment effects on NFκB change and relative power in each arm, assuming a normal distribution of the change in NFκB with a mean = 5% in the placebo arm, an equal SD = 25% in all arms, and a two sided alpha error = 5%. The primary analysis will have 80% power to detect a synergistic effect in the combination arm, which is equal to 3-fold single drug effects (i.e.: + 5% arm A vs. -55% arm D). The hypothesis of a striking synergistic effect is based on recent preclinical data of the combination of aspirin and metformin on CRC cell lines as well as pancreatic cancer cell lines [19, 20]. The secondary analyses relative to the main drug effects will have 90% power to detect a difference on the change equal to − 18.5%, compared to the placebo arm (i.e.: + 5% arm A vs. − 13.5% arm B or C), which is the same effect we detected in a previous trial with allopurinol [29]. The calculation takes into account a 10% drop-out rate: therefore the minimum number of patients to be analyzed is set to *n* = 144 (36 per arm). The sample size estimate, and relative power calculation, was performed using a simulation approach (10.000 trials) using R-project software.

**Table 3:** Hypothesized effect after 1 year of treatment on the primary endpoint

Treatment arm	N	NFκB % change	1-β (power)
Arm A (placebo)	40	+ 5%	–
Arm B (metformin)	40	−13.5%	90%
Arm C (aspirin)	40	-13.5%	90%
Arm D (aspirin+metformin)	40	−55% (~ 3.0-fold the main effects)	80%

### Statistical analysis

The synergistic effect of aspirin + metformin vs. placebo on the primary endpoint will be assessed using a two-way analysis of covariance (ANCOVA). A multivariate linear regression model will be adopted to study the change in the primary endpoint as a function of treatment and potential confounders or effect modifiers, and to adjust for imbalances between treatment arms. The interaction parameter between treatment arms will be primarily estimated and tested.

#### Secondary analyses

Two-way ANCOVA and multivariate linear modeling will also be applied to estimate and test the main effects of aspirin and metformin alone and to test the association between the secondary endpoints and treatment, adjusting for demographical and clinical factors. First and second level interaction effects between treatment and covariates (other biomarkers or demographics/clinical characteristics such as BMI and HOMA index) on primary and secondary endpoints will be tested in each model. The change of highest grade adenoma at baseline to highest grade adenoma after 1 year after randomization (no adenoma vs low/intermediate vs high grade adenoma) will be compared between the aspirin + metformin and placebo arms using a Wilcoxon test.

Gene expression levels will be analyzed using log-transformed normalized gene expression levels according to the following three-tiered approach to investigate:

- Candidate genes (e.g. *PTGS1* (*COX1*), *PTGS2* (*COX2*), *VEGF*, *TNFα*, *EGFR*, *NFκB*);
- Candidate pathways (e.g. mTOR signaling - KEGG04150, NFκB signaling - KEGG04064, VEGF signaling - KEGG04370, FoxO signaling - KEGG04068, Regulation of autophagy - KEGG04140);
- Genome-wide expression profiles.

Comparisons of changes of candidate genetic markers between arms will be assessed using linear regression from NGS data. To assess the pathway effect as part of the genetic analyses, gene-set enrichment analysis and global tests will be used. Models will be adjusted by age and gender. The effect size will be expressed using fold change values. We will visualize the possible subgroups with heat maps using unsupervised clustering. We will use Ingenuity Pathway Analysis (IPA) and Cytoscape for visualizing the results of the pathway analysis. The statistical analyses of genetic data (expression and genetic characterization of adenocarcinoma using NGS) will be performed in collaboration between the Italian and the German PIs. Statistical analyses will be performed using STATA, SAS, R-project, and Bioconductor software packages.

#### Exploratory analyses

Subpopulation Treatment-Effect Pattern Plot methodology (STEPP) [30] will be employed to display graphically the treatment effect along the continuous scale of the effect modifiers, using overlapping patient subgroups, to define subgroups of patients with different drug response.

## Discussion

Despite considerable advances in our understanding of cancer biology, early diagnosis of CRC remains elusive. Based on the adenoma-carcinoma sequence, cancer develops through the progressive accumulation of mutations in key genes that regulate cell growth. This project is the first clinical trial testing the combination of aspirin and metformin that will provide us with considerable insight into the biology of at-risk colonic mucosa adjacent to the removed neoplastic lesion. This field cancerization study could enable identification of the earliest steps in CRC recurrence and better understand the molecular targets of the chemopreventive effect of aspirin and metformin and their potentially synergistic effect in CRC patients. Moreover, the multidisciplinary approach of this study will allow communication with other researchers and departments to share ideas and foster collaborations. The trial was activated in April 2017. As of February 28, 2018, 36 patients were screened and 33 patients recruited. Recruitment is expected to end by summer of 2018 and participant treatment by summer 2019.

## Abbreviations

AE: Adverse event
AMPK: AMP kinase
bid: Bis in die
BMI: Body mass index
CRC: Colorectal cancer
CRP: C-reactive Protein
CTCAE: Common Terminology Criteria for Adverse Event
DSMB: Data and Safety Monitoring Board
HR: Hazard ratio
ICH: Immunochemistry
IGF: Insulin growth factor
IL-6: Interleukin 6
NFκB: Nuclear factor kappa-B
NGS: Next generation sequencing
NSAIDs: Nonsteroidal anti-inflammatory drugs
qd: Quaque die
SAE: Serious adverse event
SD: Standard deviation
STEPP: Subpopulation Treatment-Effect Pattern Plot
TxB2: Thromboxane B2
UWC: Umbilical waist circumference
VEGF: Vascular endothelial growth factor
